# The Use of Herbal Therapy to Improve the Quality of Life among Cancer Patients in the Southern Region of Peninsular Malaysia

**DOI:** 10.31557/APJCP.2021.22.6.1857

**Published:** 2021-06

**Authors:** Aisyah Binti Ali, Nurul Huda Razali, Neo Suk Xian, Chee Yong Sung

**Affiliations:** 1 *Clinical Research Centre Hospital Sultan Ismail, Ministry of Health Malaysia, Johor, Malaysia. *; 2 *Traditional and Complementary Unit, Hospital Sultan Ismail, Ministry of Health Malaysia, Johor, Malaysia.*

**Keywords:** Cancer, herbal therapy, quality of life (QoL)

## Abstract

**Objective:**

To investigate the impact of herbal therapy on the quality of life (QoL) among cancer patients and to evaluate the relationship of QoL with age, gender, cancer stage, cancer type, and history of conventional treatment.

**Methodology:**

A prospective study was targeted on cancer patients receiving herbal therapy from a Traditional and Complementary Medicine (T&CM) clinic in a public hospital from 1st January 2016 to 31st August 2018. The European Organization for Research and Treatment of Cancer Quality of Life Questionnaire (EORTCQLQ-C30) was distributed to the patients prior to herbal therapy (baseline) and after the sixth and twelfth week of herbal therapy. Socio-demographic and clinical data were collected and analyzed using SPSS version 16.

**Results:**

The majority of the patients were females (60.0%) and were from the Chinese ethnic group (77.4%) with a mean age of 58.72 ± 12.17 years. Approximately 42.4% of patients were in advanced cancer stages at the time of study and 60.7% of patients had undergone radiotherapy before receiving herbal therapy. The most commonly prescribed herbs were Bai Hua She She Cao (90.6%) and Zhen Ren Huo Ming Yin (57.6%). Significant differences in mean score were observed in global health status, overall functional scales, and symptom scales after the sixth and twelfth week of receiving herbal therapy. QoL in terms of global health status and overall functional scales improved with higher scores while symptom scales recorded a lower score after twelve weeks of receiving herbal therapy in the T&CM clinic. Herbal therapy has a significant effect (p < 0.05) on the improvement of QoL of cancer patients. However, gender, cancer stage, cancer type, age, history of radiotherapy, and history of chemotherapy has no effect (p > 0.05).

**Conclusion:**

Herbal therapy did improve the QoL of cancer patients in the southern region of Peninsular Malaysia.

## Introduction

Cancer patients face significant physiological, psychological, and socioeconomic challenges (Jang et al., 2017). Many patients have symptoms of general weakness, thirst, fatigue, pain, diarrhea, nausea, and vomiting during or after chemotherapy or radiotherapy (Darus, 2016). Emotional disturbances such as anxiety and depression are also common during diagnosis and treatment (Jang et al., 2017). These symptoms and disturbances potentially contribute to significant negative impacts on their quality of life (QoL) as patients often delay or reduce the extent of chemotherapy (Al-Naggar et al., 2013). Reducing symptom burden caused by cancer itself or cancer-related treatment is high on the clinical research agenda (Tishelman et al., 2007; Akin et al., 2010) as such burden is negatively correlated to the QoL of cancer patients (Steele et al., 2005; Akin et al., 2010). In advanced or recurrent cancers, the goal of cancer treatment is usually not curing the disease but prolonging survival time with good QoL. QoL is valued as a cancer outcome, and the effect of early palliative care is emphasized importantly (Temel et al., 2010).

There is a growing interest in complementary and alternative medicine (CAM) in Eastern countries (Choi et al., 2012; Jang et al., 2017; Chun et al., 2017; Razali et al., 2020). Up to 50% of cancer patients in the Asian population are known to use CAM (Shih et al., 2009; Kang et al., 2012). Similarly, previous study reported that 62% of cancer patients in the southern region of Peninsular Malaysia use CAM (Razali et al., 2020). The use of herbal medicine has been high among Asian cancer patients (Cui et al., 2004; Kim et al., 2013; Kalender et al., 2014). Similar reports are found in Malaysia in which biologically based therapies such as herbs and dietary supplements (87.9%) are the most frequent type of CAM used by cancer patients in South Peninsular Malaysia (Razali et al., 2020).

Traditional Chinese Medicine (TCM) is commonly used among the general and sick population for various reasons and purposes (Kang et al., 2014; Naja et al., 2015; Stussman et al., 2015) and up to 79% of cancer patients use TCM (Kang et al., 2012; Choi et al., 2012; John et al., 2016). Chinese Herbal Medicine (CHM), the major component of TCM, has been increasing in popularity among cancer patients (Cho, 2012). High use of CHM is due to the belief that TCM helps to improve therapeutic outcomes and QoL among cancer patients (Hahoshen et al., 2011). In Malaysia, the overall prevalence of ever used CHM with and without consultation of qualified Chinese Medicine practitioners among cancer patients are 44.1% and 55.9% respectively (Loke et al., 2017).

The government of Malaysia has acknowledged integrative treatment of Traditional and Complementary (T&CM) into the modern healthcare system. This in line with the emphasis of the World Health Organization (WHO) on the importance of T&CM in overall health care management through the Regional Strategy for Traditional Medicine in the Western Pacific (2011-2020) and the WHO Traditional Medicine Strategy (2014-2023). One of such integrative treatment strategies was to establish public T&CM practices in selected government hospitals (Hamid et al., 2011). In Malaysia, herbal therapy treatments are available at four Ministry of Health hospitals namely National Cancer Institute (Putrajaya), Hospital Kepala Batas (Pulau Pinang), Hospital Sultan Ismail (Johor Bahru), and Hospital Wanita & Kanak-Kanak Sabah. A guideline was developed to document the practice of using T&CM on herbal therapy as an adjunct treatment for cancer (Soon et al., 2018). As a standard of practice, the guideline outlined that all cancer patients seeking adjunct treatment at T&CM units should be referred by a medical oncologist after proper investigation and precise diagnosis have been made. A qualified herbal practitioner will then provide consultation and prescribe herbal treatment (Soon et al., 2018). A study has reported that approximately 19% of cancer patients utilize TCM during chemotherapy in Malaysia (Chui et al., 2014). It is irrefutable that herbal therapy has the potential to effectively support therapeutic care while improving QoL.

Nevertheless, to date, there is a lack of evidence on the QoL of cancer patients who received herbal therapy in Malaysia. Therefore, in this study, we explored the effect of herbal therapy on the QoL of cancer patients in public T&CM clinics in Malaysia.

## Materials and Methods

*Study design*

A longitudinal study from January 2016 till December 2018 was conducted in T&CM Clinic, Hospital Sultan Ismail, Johor.

*Ethics*

Approval from the Medical Research Ethics Committee, Ministry of Health Malaysia was obtained before the commencement of the study with approval number of NMRR-15-1065-26327. Written informed consent was obtained from all participants who agreed to participate. No ethical problems were encountered during the study.

*Participants and sample size calculation*

Participants consisted of cancer patients referred from the Oncology Clinic, Hospital Sultan Ismail who sought herbal therapy at the T&CM clinic of Hospital Sultan Ismail. The majority of the referred patients were in advanced cancer stages and had already failed oncology treatment. The eligibility and recruitment of patients were determined by TCM practitioners of the T&CM clinic. Patients aged above 18 with confirmed diagnosis of cancer regardless of tumor type and had not received any concurrent medical treatment were included. On the other hand, patients with abnormal renal or liver function, with heart failure or requiring oxygen therapy, and patients who were either pregnant or breastfeeding, were taking other alternative medicine or supplements, or were medically unstable, were excluded. The sample size was calculated using Power & Sample Size Program, Dupont and Plummer, 1997 in which 85 patients were required to detect group differences at 5% with a 95% confidence level and 80% power of study. A convenient sampling method was applied to obtain the samples.

*Outcomes measured*

The primary outcome of this study was the QoL of participants comprising of global health status (GHS) score, functional score, and symptom score of the European Organization for Research and Treatment of Cancer Quality of Life Questionnaire (EORTC QLQ-C30). The QoL of participants was assessed at the baseline, at Week 6, and at Week 12.

*Study instrument*

The interviews were conducted in Malay, English and Mandarin Chinese. Patients were guided by the interviewer to avoid misunderstanding. The questionnaires consisted of four sections: Section A, Section B, Section C, and Section D. Section A was designed to collect socio-demographic information and Section B was intended to include the medical history of the patients. Section C was comprised of the three components namely GHS/QoL score, functional score, and symptom score from the EORTC QLQ-C30 (Aaronson et al., 1993).

The scoring procedure was based on The EORTC QLQ-C30 Scoring Manual (3^rd^ Edition) provided by the EORTC Quality of Life Group (Fayers et al., 2001). The QLQ-C30 version 1.0 (QLQ-C30(V1)) incorporated five functional scales (physical, role, cognitive, emotional, and social), three symptom scales (fatigue, pain, and nausea and vomiting), a GHS/QoL scale, a number of single items assessing additional symptoms commonly reported by cancer patients (dyspnoea, loss of appetite, insomnia, constipation, and diarrhea), and perceived financial impact of the disease.

For all scales, the raw score (RS) is the mean of the component items: RS = (I1 + I2 +...+n)/ n. For functional scales, Score = {1- (RS - 1)/ range*} x 100. For symptom

and GHS scales, Score = {(RS - 1)/ range*} x 100. *range is the difference between the maximum possible value and minimum possible value (Fayers et al., 2001). All scales and single-item measurements range from 0 to 100. A high scale score represents a high response level. Thus, i) a high score of the functional scale represents a high/healthy level of functioning, ii) a high score of the GHS/QoL scale represents a high QoL, but iii) a high score of the symptom scale/item represents a high level of symptomatology/problems. The raw score was calculated by estimating the average of the items which contributed to the scale. Transformation was then performed to standardize the raw scores (Fayers et al., 2001).

*Data collection*

A baseline assessment (V1) of QoL was made after obtaining written consent. Patients were then given herbal medicine therapy containing Chinese herbs that had been approved by the Ministry of Health, Malaysia, according to the prescription of the herbal oncologist in the T&CM clinic. Post-test assessment of the QoL was made at the sixth week (V2) and twelfth week (V3) of herbal therapy. Patients were asked to keep a diary recording their drug compliance and these diaries were collected at these two points. 

*Data analysis*

Data was entered, cleaned, and analyzed using the PASW Statistics® 18 © 2009 Statistical Packages for Social Science (SPSS), version 16.0, IBM Corporation, (New York, USA). Mean and standard deviation (sd) were used to describe the characteristics of the patients for continuous data, whereas percentage was used for categorical data. The outcome measurement on QoL between pre-treatment, sixth week of treatment, and twelfth week of treatment were compared using general linear model repeated measure test. The relationship between QoL with patient demographic and clinical characteristics were analyzed using repeated measures ANOVA. A P value of less than 0.05 is considered statistically significant.

## Results

*Clinical characteristics*

**Figure 1:**
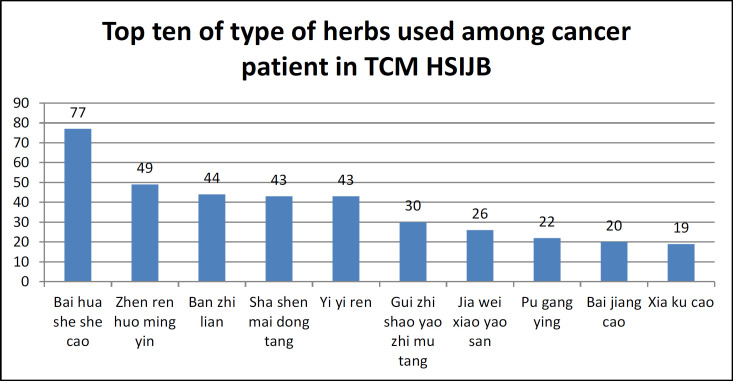
Top Ten of Type of Herbs Used among Cancer Patients in TCM HSIJB

A total of 85 eligible cancer patients from the T&CM clinic of Hospital Sultan Ismail, Johor were enrolled in the study after obtaining consent. The mean age ± standard deviation of the participants was 58.72 ± 12.17 years. The majority of the participants were female (60.0%), Chinese (77.4%), Buddhist (69.9%), and had secondary level of education (51.3%) (Table 1). The most frequent diagnosis was gastrointestinal tract (GIT) cancer (30.6%) and the least was lung (8.2%) and gynecological cancer (8.2%). A total of 36 (42.4%) advanced cancer stages were reported at the time of the study. In addition, the majority of the patients had undergone radiotherapy (60.7%) before receiving herbal therapy in the T&CM clinic (Table 2). Figure 1 shows the top ten types of herbs used by cancer patients with the most used herb among cancer patients being Bai Hua She She Cao (90.6%), followed by Zhen Ren Huo Ming Yin (57.6%) and the least used herb being Xia Ku Cao (22.4%).

QoL scores different between assessment visits The mean (standard deviation) scores of GHS increased from 54.31 (15.78) to 70.20 (14.05) after 12 weeks of receiving herbal therapy (Table 3). Each subscale of functional scores also increased in mean (standard deviation) from 47.35 (20.90) to 84.31 (19.13) after 12 weeks of receiving herbal therapy (Table 3). The mean (standard deviation) scores of symptom scales decreased from 62.74 (15.78) to 8.82 (19.69) after 12 weeks of receiving herbal therapy (Table 3).

A repeated measures ANOVA with a Greenhouse-Geisser correction showed that the mean value of score of GHS scales, functional subscales, and symptom subscales were statistically significant between assessment stages (baseline, 6 weeks, and 12 weeks of herbal therapy) (p < 0.001). Post hoc comparison test using the Bonferroni correction revealed that the mean scores of GHS scales, functional subscales, and symptom subscales between baseline and 6 weeks of herbal therapy, baseline and 12 weeks of herbal therapy, and also between 6 weeks and 12 weeks of herbal therapy were significantly different (Table 4) 

*Relationship between QoL scores with risk factors*

Results found that GHS scales, functional subscales and symptom subscales (baseline, 6 weeks of herbal therapy, and 12 weeks of herbal therapy) are significant (p < 0.001), indicating that herbal therapy is effective in increasing GHS and functional subscales score, and reducing symptom subscales score. However, gender, cancer stage, cancer type, age, history of radiotherapy, and history of chemotherapy do not show significant effect (p > 0.05) in increasing GHS and functional subscales score, and reducing symptom subscales score (Table 4).

**Table 1:** Socio-Demographic Characteristics of the Subjects (n=85)

Socio-demographic	n (%)	
Age (years)	58.7	12.17
[mean (SD)]		
Gender		
Female	51	60.0
Male	34	40.0
Education level		
Primary	27	38.8
Secondary	41	51.3
University	12	15.0
Race		
Malay	18	21.4
Chinese	65	77.4
Indian	1	1.2
Religion		
Islam	19	22.9
Buddhist	58	69.9
Christian	4	4.8
Others	2	2.4

**Table 2:** Clinical Characteristics of the Subjects (n=85)

Socio-demographic	n (%)	
Cancer stage		
Stage 1	8	9.4
Stage 2	6	7.1
Stage 3	8	9.4
Stage 4	36	42.4
Unknown	27	31.8
Cancer type		
Breast	16	18.8
GIT	26	30.6
Gynecological	7	8.2
Lung	7	8.2
NPC	8	9.4
Others	10	11.8
Hematology	11	12.9
History of radiotherapy		
Yes	51	60.7
No	31	39.3
History of chemotherapy		
Yes	17	20.2
No	27	79.8
History of surgery		
Yes	41	48.8
No	43	51.2

**Table 3:** Quality of Life (EORTC QLQ-C30) Score According to Domains Reported in Baseline, 6 Weeks and 12 Weeks after Received Herbal Medicine Therapy

EORTC QLQ-C30	Mean QoL score ± SD		
	Baseline	6 weeks after treatment	12 weeks after treatment
Global health status	54.31 (15.78)	63.23 (12.15)	70.20 (14.05)
Functional scales			
Physical	51.68 (19.73)	67.45 (21.51)	80.39 (20.25)
Role	45.68 (26.87)	62.74 (26.31)	75.88 (24.60)
Emotional	47.35 (20.09)	66.76 (18.39)	77.84 (19.01)
Cognitive	47.84 (19.89)	69.21 (18.63)	81.17 (16.02)
Social	52.94 (21.85)	71.37 (19.18)	84.31 (19.13)
Symptom scales			
Fatigue	62.74 (15.78)	39.86 (16.74)	32.67 (20.82)
Nausea & vomitting	49.80 (28.23)	23.33 (22.89)	8.82 (19.69)
Pain	60.39 (22.99)	38.43 (21.21)	28.43 (22.83)
Dyspnoea	50.98 (26.52)	30.19 (23.36)	21.56 (24.51)
Insomnia	57.25 (27.98)	36.86 (23.58)	25.09 (28.13)
Appetite loss	42.74 (25.52)	25.09 (23.52)	15.29 (24.97)
Constipation	50.19 (23.92)	20.78 (22.41)	12.54 (22.41)
Diarrhea	39.21 (24.77)	20.39 (19.98)	10.19 (17.08)
Financial difficulties	49.80 (26.04)	31.76 (21.15)	16.47 (20.97)

**Table 4:** Overall Significant Difference between Means of QoL of Cancer Patients after Receiving Herbal Therapy at a Different Time Point

Source	F statistic (df, std. error)	Effect size	p-value
Global health status	57.797 (1.724, 144.784)	0.408	<0.001
Functional scales			
Physical functioning	83.546 (2, 168)	0.499	<0.001
Role functioning	65.092 (2, 168)	0.437	<0.001
Emotional functioning	78.135 (1.703, 143.049)	0.482	<0.001
Cognitive functioning	90.781 (1.898, 159.428)	0.519	<0.001
Social funtioning	68.259 (2, 168)	0.448	<0.001
Symptoms scales			
Fatigue	102.095 (2, 168)	0.549	<0.001
Nausea and vomitting	86.114 (2, 168)	0.506	<0.001
Pain	74.782 (2, 168)	0.471	<0.001
Dyspnoea	52.747 (2, 168)	0.386	<0.001
Insomnia	47.584 (1.783, 149.766)	0.362	<0.001
Appetite loss	44.103 (1.857, 156.024)	0.344	<0.001
Constipation	117.791 (1.903, 159.88)	0.584	<0.001
Diarrhoea	57.824 (2, 168)	0.408	<0.001
Financial difficulties	67.847 (2, 168)	0.447	<0.001

Repeated measure ANOVA. A P value of < 0.05 is considered statistically significant

## Discussion

To our knowledge, this is one of the first few studies to report on the improvement of the QoL of cancer patients who had received herbal therapy from T&CM clinics in Malaysian government hospitals. Based on our findings, females and older patients tend to use herbal therapy more than males and younger patients, similar to findings reported in several previous studies (Chun et al., 2017; Loke et al., 2017). McLauglin (2012) reported that the higher prevalence of CAM usage in the older age group is attributed by a push factor which includes dissatisfaction with conventional health services and a pull factor related to positive traits of selection and self-care. Previous studies have reported that patients in advanced stages of diseases are more prone to use herbal therapy (Liu et al., 2012; P. Puataweepong et al., 2012). Similar results were obtained in our study. As these patients have little hope in conventional treatments and often experience serious adverse effects from conventional therapies, they turn to herbal therapy as an alternative intervention to improve the quality of their lives (Liu et al., 2012).

WHO defines herbal medicine as plant-derived materials (which contain either raw or processed ingredients from one or more plants), or preparation used for treatment or other human health benefits (World Health Organization Regional Office for the Western Pacific, 1998). Our study found that Bai Hua She She Cao was the most commonly prescribed herb, followed by Zhen Ren Huo Ming Yin, Ban Zhi Lian, She Shen Mai Dong Tang, and Yi Yi Ren. This was different when compared with a study by Loke (2017) which found that Chinese herbal extract (27%), Sabah snake grass (12.2%), and Da Shuan (ginger) (11.5%) were the herbs of choice by their population. Only 4.6% of their population used Bai Hua She She Chao. This difference may occur as our study only focused on patients who consulted a qualified Chinese Medicine practitioner after diagnosis was made, whereas Loke (2017) reported that half of the herbs used were self-prescribed without consulting a qualified practitioner. Bai Hua She She Cao is a herb derived from the whole plant of Hedyotis diffusa willd in the family Rubiaceae (Rui et al., 2016). It has solitary flowers on thick and short pedicles (2-4 mm in length) growing from cylindrical stems (Flora Republic Plaparis Sinicae). It is a well-known plant used to treat hepatitis, tonsillitis, urethral infection, and malignant tumors of the liver and lung. Previous study has reported multiple biological activities of the Oldenlandia Diffusa (OD) such as antitumor, chemo-preventive, anti-inflammatory, antioxidant, and proapoptotic effects (Ganbold et al., 2010; Gu et al., 2012). One in vivo study using the chemically induced liver cancer model by Sunwoo (2012) noted that OD has an anticancer effect in caspase-3 induced apoptosis, antiproliferation effect, and inhibition of migration through its bioactive component: oleanolic acid and ursolic acid. 

Our study indicated that the QoL of cancer patients significantly improved after receiving herbal therapy. QoL among cancer patients was better with respect to the GHS, showing high scores in overall functioning and low scores in symptoms after receiving herbal therapy for 6 weeks and 12 weeks. This finding was consistent with the aims of herbal therapy used as an adjunct therapy in cancer treatment to reduce cancer symptoms and complications, minimize side effects from conventional cancer treatment, improve body immune system, provide synergistic effects, and to improve QoL of patients (Soon et al., 2018). In contrast, Loke (2017) reported no significant difference when comparing the QoL between users and non-users of herbal medicines. Stewart (2017) conducted a meta-analysis and concluded that it is unclear whether herbal remedies can have a beneficial impact on the QoL of cancer patients. Another study demonstrates that herbal medicine used among the Chinese population in Hong Kong does not result in an overall improvement in the QoL of cancer patients, even though herbal medicine positively affects the body’s immune system (Chan et al., 2015). Except for herbal therapy, the present study showed that other factors have no significant effect on the increase of GHS and functional subscales score, and the reduction of the symptom subscales score. Although there is still much debate about the efficacy of herbal medicines, more data are demonstrating that herbal medicines have the potential to improve tumor response to chemotherapy as well as patient survival rates (Shu et al., 2005). Furthermore, Li (2017) who conducted a meta-analysis on herbal medicines and its effect on the QoL found that herbal medicines have beneficial effects on the QoL of cancer patients. 

Our study has some limitations. Since our researchers were mostly of the Malay ethnic group, the convenient sampling method coupled with face-to-face interview might have exposed the patients to a selection bias as the Chinese and Indians who were not well versed in the language might decline their participations due to language barriers. On some occasions, the face-to-face interview might have made participants uncomfortable to disclose certain information due to the fear of receiving negative perceptions. In this study, although we reported that more than 50% of the patients were given Bai Hua She She Cao by registered TCM practitioners, the study only focused on one major government hospital based in the southern area. It is unclear if similar findings will be yielded from registered TCM practices from other government hospitals. More studies on specific cancer in specific herbal therapy should be conducted in the future to provide a clear and precise outcome of receiving herbal therapy. Other than that, further multicenter studies should be conducted to explore the herbal therapy used among health professionals of registered TCM practices in some of the hospitals in Malaysia.

In conclusion, our study indicated that herbal therapy is effective in increasing the GHS and functional subscales score, and reducing symptom subscales score. Thus, herbal therapy has been proven to improve the QoL among cancer patients admitted to the government hospitals in the southern region of Malaysia. 

## Author Contribution Statement

AA was involved in the conception and design of the study, analysis of data, interpretation of results, and initial drafting of the manuscript. NHR was involved in the conception and design of the study and drafting of the manuscript. NSX and CYS were involved in the conception and design of the study, collection of data, and interpretation of results. All authors read and approved the final manuscript.
